# Development and validation of nomograms to predict early death for elderly lung cancer patients

**DOI:** 10.3389/fsurg.2023.1113863

**Published:** 2023-02-13

**Authors:** Jiafei Li, Qian Zou, Rubing Gu, Fang Wang, Xun Li

**Affiliations:** Department of Respiratory and Critical Care, Medicine, Chuzhou Hospital of Anhui Medical University, Anhui, China

**Keywords:** early death, lung cancer, nomogram, risk factor, SEER database

## Abstract

**Background:**

Due to the aging of society, the average age of LC (lung cancer) patients has increased in recent years. The purpose of this study was to determine the risk factors and develop nomograms to predict the probability of early death (dead in three months) for elderly (≥ 75 years old) LC patients.

**Methods:**

Data of elderly LC patients were obtained from the SEER database by using the SEER stat software. All patients were randomly divided into a training cohort and a validation cohort in a ratio of 7:3. The risk factors of all-cause early and cancer-specific early death were identified by univariate logistic regression and backward stepwise multivariable logistic regression in the training cohort. Then, risk factors were used to construct nomograms. The performance of nomograms was validated by receiver operating curves (ROC), calibration curves, and decision curve analysis (DCA) in the training cohort and validation cohort.

**Results:**

A total of 15,057 elderly LC patients in the SEER database were included in this research and randomly divided into a training cohort (*n* = 10,541) and a validation cohort (*n* = 4516). The multivariable logistic regression models found that there were 12 independent risk factors for the all-cause early death and 11 independent risk factors for the cancer-specific early death of the elderly LC patients, which were then integrated into the nomograms. The ROC indicated that the nomograms exhibited high discriminative ability in predicting all-cause early (AUC in training cohort = 0.817, AUC in validation cohort = 0.821) and cancer-specific early death (AUC in training cohort = 0.824, AUC in validation cohort = 0.827). The calibration plots of the nomograms were close to the diagonal line revealing that there was good concordance between the predicted and practical early death probability in the training and validation cohort. Moreover, the results of DCA analysis indicated that the nomograms had good clinical utility in predicting early death probability.

**Conclusion:**

The nomograms were constructed and validated to predict the early death probability of elderly LC patients based on the SEER database. The nomograms were expected to have high predictive ability and good clinical utility, which may help oncologists develop better treatment strategies.

## Introduction

Lung cancer (LC) is one of the most common malignant tumors and causes of cancer-related death in the United States, with an estimated 2.3 million newly diagnosed patients and 1.8 million deaths in 2020 (1). With the rapid development of diagnostic techniques and increased awareness of health examinations, the early detection rate of LC has improved sharply. The prognosis of LC is related to the American Joint Committee on Cancer (AJCC) tumor-node-metastasis (TNM) staging system (2). However, the AJCC staging system does incorporate tumor size, lymph nodes, and metastases, but neglects patient heterogeneity (3). Therefore, a more comprehensive and accurate prognostic model is needed.

The average age of cancer patients has gradually increased due to the rapid aging of the global population (4). According to a single-institution study, the systemic therapy rate in elderly LC patients was significantly lower than in younger patients (5). According to a recent study, researchers found that elderly LC patients have a higher early mortality rate (6). Besides that, recent research found that the mortality (8.2 vs. 2.2%) and postoperative problems (26.0 vs. 13.3%) increased significantly in elderly LC patients when compared with younger patients (7). Moreover, the research found that about 70% of LC patients and more than 70% of LC deaths occurred in elderly LC patients (8). Early death is defined as death accorded within three months after diagnosis (6). A deep understanding of the relationship between early death and elderly LC risk factors may help physicians improve the elderly patients' survival and life quality and provide individualized treatment strategies.

The Surveillance, Epidemiology, and End Results (SEER) database is a large-scale database that covers approximately 35% of the United States. population. Nomogram is a tool for precisely predicting cancer patient outcomes that can provide personalized risk estimates (9). In this research, we identified the risk factors of elderly LC patients and constructed and validated the nomograms for predicting the risk probability of elderly LC patients. The nomograms may play an important role in identifying high-risk patients, selecting treatment strategies, and managing follow-up.

## Methods

### Patients and methods

Clinicopathological information of elderly (≥ 75 years old) LC patients from 2010 to 2015 was extracted from the dataset of “Incidence-SEER Research Plus Data, 18 Registries, Nov 2020 Sub (2010–2018)” in the SEER database. The SEER stat software was used to obtain the clinicopathological information (version 8.4.0; http://seer.cancer.gov/seerstat/). The endpoint of this research was all-cause early death and cancer-specific early death. All-cause early death was defined as patients dead in three months because of any cause, and cancer-specific early death was regarded as the time from diagnosis to death due to LC less than three months.

The inclusion criteria were as follows: (1) Age ≥75; (2) Site recode ICD-O-3/WHO 2008: lung and bronchus; (3) patients were one primary tumor only; (4) patients were positive histology confirmed; (5) Patients with complete and clear data on indicators, including sex, race, marital status, laterality, primary site, Grade, AJCC stage, T, N, M, surgery, radiation, chemotherapy, bone metastasis, brain metastasis, pathological type, liver metastasis, survival time and survival state. All patients included in this study were divided into a training cohort and a validation cohort in a ratio of 7:3.

### Nomograms development

The univariate logistic regression models of all-cause early and cancer-specific early death contained 17 potential factors. Significantly variables with *p* < 0.05 in univariate logistic regression models were then assessed by backward stepwise multivariable logistic regression in the training cohort. Based on the independent prognostic factors identified by multivariable logistic regression, predictive nomograms were constructed in the training cohort.

### Nomograms validation

The training and validation cohorts were used to validate the value of the nomograms. ROC curves were adopted as the main index of discriminative power, the area under the ROC curves (AUC) ranges from 0.5 to 1.0, and the higher the AUC, the more perfect the discrimination value. Moreover, calibration curves were used to verify the accuracy and reliability of nomograms, and DCA curves were utilized to measure the clinical practice of nomograms.

### Statistical analysis

Categorical variables were compared by Chi-squared test and described by Number and percentage (n, %), while quantitative data were compared by t-test and described by mean ± standard deviation (SD). All analyses were performed by R software (version 4.0.2), and the *p*-value (two-tail) < 0.05 was regarded as statistical significance.

## Results

### Demographic and clinical characteristics

As shown in Table 1, a total of 15,057 elderly LC patients were enrolled in this research, 4,549 cases were all-cause early death, and 3,977 cases were cancer-specific early death. Of these patients, 52.6% were male. In the primary site subgroup, most patients were upper lobe LC (58.8%), with middle lobe (4.6%), lower lobe (35.5%), and overlapping (1.2%). Most of the patients were in Grade III (51.5%) and AJCC stage IV (36.5%). In the treatment subgroup, 26.1% (3929) of the patients received surgery, 38.9% (5857) received radiotherapy, and 32.5% (4887) received chemotherapy respectively. Overall, 12.7% (1919), 7.1% (1074), and 6.4% (958), of 15,057 elderly LC patients had bone, brain, liver metastases, respectively.

**Table 1:** Demographic and clinicopathological characteristics of elderly LC patients in the SEER database.

	Characteristics	Overall (*n* = 15,057)	No early death (*n* = 10,508)	All-cause early death (*n* = 4549)	Cancer-specific early death (*n* = 3977)
Sex (%)	Male	7,923 (52.6)	5,312 (50.6)	2,611 (57.4)	2,267 (57.0)
	Female	7,134 (47.4)	5,196 (49.4)	1,938 (42.6)	1,710 (43.0)
Race (%)	White	12,650 (84.0)	8,866 (84.4)	3,784 (83.2)	3,317 (83.4)
	Black	1,258 (8.4)	843 (8.0)	415 (9.1)	354 (8.9)
	Other	1,149 (7.6)	799 (7.6)	350 (7.7)	306 (7.7)
Marital status (%)	Married	7,483 (49.7)	5,301 (50.4)	2,182 (48.0)	1,913 (48.1)
	Unmarried	7,574 (50.3)	5,207 (49.6)	2,367 (52.0)	2,064 (51.9)
Laterality (%)	Right	8,737 (58.0)	6,062 (57.7)	2,675 (58.8)	2,308 (58.0)
	Left	6,317 (42.0)	4,445 (42.3)	1,872 (41.2)	1,667 (41.9)
	Paired site	3 (0.0)	1 (0.0)	2 (0.0)	2 (0.1)
Primary Site (%)	Upper lobe	8,847 (58.8)	6,165 (58.7)	2,682 (59.0)	2,345 (59.0)
	Middle lobe	686 (4.6)	501 (4.8)	185 (4.1)	166 (4.2)
	Lower lobe	5,345 (35.5)	3,740 (35.6)	1,605 (35.3)	1,397 (35.1)
	Overlapping	179 (1.2)	102 (1.0)	77 (1.7)	69 (1.7)
Grade (%)	I	1,328 (8.8)	1,083 (10.3)	245 (5.4)	214 (5.4)
	II	5,170 (34.3)	3,946 (37.6)	1,224 (26.9)	1,017 (25.6)
	III	7,755 (51.5)	5,026 (47.8)	2,729 (60.0)	2,434 (61.2)
	IV	804 (5.3)	453 (4.3)	351 (7.7)	312 (7.8)
AJCC Stage (%)	I	4,384 (29.1)	3,812 (36.3)	572 (12.6)	384 (9.7)
	II	1,015 (6.7)	837 (8.0)	178 (3.9)	138 (3.5)
	III	4,167 (27.7)	3,001 (28.6)	1,166 (25.6)	1,012 (25.4)
	IV	5,491 (36.5)	2,858 (27.2)	2,633 (57.9)	2,443 (61.4)
T (%)	T1	2,954 (19.6)	2,525 (24.0)	429 (9.4)	308 (7.7)
	T2	6,162 (40.9)	4,535 (43.2)	1,627 (35.8)	1,413 (35.5)
	T3	1,023 (6.8)	688 (6.5)	335 (7.4)	302 (7.6)
	T4	4,918 (32.7)	2,760 (26.3)	2,158 (47.4)	1,954 (49.1)
N (%)	N0	7,149 (47.5)	5,570 (53.0)	1,579 (34.7)	1,294 (32.5)
	N1	1,483 (9.8)	1,039 (9.9)	444 (9.8)	377 (9.5)
	N2	5,151 (34.2)	3,130 (29.8)	2,021 (44.4)	1,836 (46.2)
	N3	1,274 (8.5)	769 (7.3)	505 (11.1)	470 (11.8)
M (%)	M0	9,566 (63.5)	7,650 (72.8)	1,916 (42.1)	1,534 (38.6)
	M1	5,491 (36.5)	2,858 (27.2)	2,633 (57.9)	2,443 (61.4)
Surgery (%)	No	11,128 (73.9)	7,077 (67.3)	4,051 (89.1)	3,634 (91.4)
	Yes	3,929 (26.1)	3,431 (32.7)	498 (10.9)	343 (8.6)
Radiation (%)	No/unknown	9,200 (61.1)	5,907 (56.2)	3,293 (72.4)	2,820 (70.9)
	Yes	5,857 (38.9)	4,601 (43.8)	1,256 (27.6)	1,157 (29.1)
Chemotherapy (%)	No/unknown	10,170 (67.5)	6,428 (61.2)	3,742 (82.3)	3,250 (81.7)
	Yes	4,887 (32.5)	4,080 (38.8)	807 (17.7)	727 (18.3)
Bone metastasis (%)	No	13,138 (87.3)	9,605 (91.4)	3,533 (77.7)	3,010 (75.7)
	Yes	1,919 (12.7)	903 (8.6)	1,016 (22.3)	967 (24.3)
Brain metastasis (%)	No	13,983 (92.9)	10,069 (95.8)	3,914 (86.0)	3,369 (84.7)
	Yes	1,074 (7.1)	439 (4.2)	635 (14.0)	608 (15.3)
Liver metastasis (%)	No	14,099 (93.6)	10,109 (96.2)	3,990 (87.7)	3,452 (86.8)
	Yes	958 (6.4)	399 (3.8)	559 (12.3)	525 (13.2)
Pathological type (%)	NSCLC	13,750 (91.3)	9,691 (92.2)	4,059 (89.2)	3,535 (88.9)
	SCLC	1,307 (8.7)	817 (7.8)	490 (10.8)	442 (11.1)

As shown in Table 2, a total of 15,057 patients were randomly divided into the training cohort (*n* = 10,541) and the validation cohort (*n* = 4516). Results revealed that there has no statistically significant difference between the training cohort and the validation cohort in sex (*p* = 0.995), race (*p* = 0.674), marital status (*p* = 0.855), laterality (*p* = 0.524), primary site (*p* = 0.269), Grade (*p* = 0.074), AJCC stage (*p* = 0.817), T (*p* = 0.584), N (*p* = 0.73), M (*p* = 0.668), surgery (*p* = 0.653), radiation (*p* = 0.378), chemotherapy (*p* = 0.739), bone metastasis (*p* = 0.079), brain metastasis (*p* = 0.802), liver metastasis (*p* = 0.504), pathological type (*p* = 0.269).

**Table 2:** The baseline characteristics of the training and validation cohorts.

	Characteristics	Overall (*n* = 15,057)	Training cohort (*n* = 10,541)	Validation cohort (*n* = 4516)	*p*
Sex (%)	Male	7,923 (52.6)	5,546 (52.6)	2,377 (52.6)	0.995
	Female	7,134 (47.4)	4,995 (47.4)	2,139 (47.4)
Race (%)	White	12,650 (84.0)	8,852 (84.0)	3,798 (84.1)	0.674
	Black	1,258 (8.4)	873 (8.3)	385 (8.5)	
	Other	1,149 (7.6)	816 (7.7)	333 (7.4)	
Marital status (%)	1	7,483 (49.7)	5,233 (49.6)	2,250 (49.8)	0.855
	2	7,574 (50.3)	5,308 (50.4)	2,266 (50.2)
Laterality (%)	Right	8,737 (58.0)	6,114 (58.0)	2,623 (58.1)	0.524
	Left	6,317 (42.0)	4,424 (42.0)	1,893 (41.9)
	Paired site	3 (0.0)	3 (0.0)	0 (0.0)	
Primary Site (%)	Upper lobe	8,847 (58.8)	6,246 (59.3)	2,601 (57.6)	0.269
	Middle lobe	686 (4.6)	481 (4.6)	205 (4.5)	
	Lower lobe	5,345 (35.5)	3,691 (35.0)	1,654 (36.6)
	Overlapping	179 (1.2)	123 (1.2)	56 (1.2)	
Grade (%)	I	1,328 (8.8)	926 (8.8)	402 (8.9)	0.074
	II	5,170 (34.3)	3,602 (34.2)	1,568 (34.7)
	III	7,755 (51.5)	5,417 (51.4)	2,338 (51.8)
	IV	804 (5.3)	596 (5.7)	208 (4.6)	
AJCC Stage (%)	I	4,384 (29.1)	3,093 (29.3)	1,291 (28.6)	0.817
	II	1,015 (6.7)	705 (6.7)	310 (6.9)	
	III	4,167 (27.7)	2,911 (27.6)	1,256 (27.8)
	IV	5,491 (36.5)	3,832 (36.4)	1,659 (36.7)
T (%)	T1	2,954 (19.6)	2,075 (19.7)	879 (19.5)	0.584
	T2	6,162 (40.9)	4,342 (41.2)	1,820 (40.3)
	T3	1,023 (6.8)	717 (6.8)	306 (6.8)	
	T4	4,918 (32.7)	3,407 (32.3)	1,511 (33.5)
N (%)	N0	7,149 (47.5)	5,012 (47.5)	2,137 (47.3)	0.73
	N1	1,483 (9.8)	1,021 (9.7)	462 (10.2)	
	N2	5,151 (34.2)	3,607 (34.2)	1,544 (34.2)
	N3	1,274 (8.5)	901 (8.5)	373 (8.3)	
M (%)	M0	9,566 (63.5)	6,709 (63.6)	2,857 (63.3)	0.668
	M1	5,491 (36.5)	3,832 (36.4)	1,659 (36.7)
Surgery (%)	No	11,128 (73.9)	7,802 (74.0)	3,326 (73.6)	0.653
	Yes	3,929 (26.1)	2,739 (26.0)	1,190 (26.4)
Radiation (%)	No/unknown	9,200 (61.1)	6,416 (60.9)	2,784 (61.6)	0.378
	Yes	5,857 (38.9)	4,125 (39.1)	1,732 (38.4)
Chemotherapy (%)	No/unknown	10,170 (67.5)	7,129 (67.6)	3,041 (67.3)	0.739
	Yes	4,887 (32.5)	3,412 (32.4)	1,475 (32.7)
Bone metastasis (%)	No	13,138 (87.3)	9,231 (87.6)	3,907 (86.5)	0.079
	Yes	1,919 (12.7)	1,310 (12.4)	609 (13.5)	
Brain metastasis (%)	No	13,983 (92.9)	9,785 (92.8)	4,198 (93.0)	0.802
	Yes	1,074 (7.1)	756 (7.2)	318 (7.0)	
Liver metastasis (%)	No	14,099 (93.6)	9,880 (93.7)	4,219 (93.4)	0.504
	Yes	958 (6.4)	661 (6.3)	297 (6.6)	
Pathological type (%)	NSCLC	13,750 (91.3)	9,608 (91.1)	4,142 (91.7)	0.269
	SCLC	1,307 (8.7)	933 (8.9)	374 (8.3)	

### Univariate and multivariate logistic analysis in the training cohort

In order to screen the prognostic factors in elderly LC patients, univariate and multivariate logistic analyses in the training cohort were simultaneously conducted. According to univariate regression analysis (Table 3), variables including sex, race, marital status, primary site, Grade, AJCC stage, T, N, M, surgery, radiation, chemotherapy, bone metastasis, brain metastasis, liver metastasis, and pathological type were significantly associated with all-cause early and cancer-specific early death, with all *p* < 0.05. Moreover, the backward stepwise multivariate logistic analysis revealed that sex, primary site, grade, AJCC stage, T, N, surgery, radiation, chemotherapy, bone metastasis, brain metastasis, and liver metastasis were independent prognostic factors for all-cause early death, while sex, grade, AJCC stage, T, N, surgery, radiation, chemotherapy, bone metastasis, brain metastasis, and liver metastasis were independent prognostic factors for cancer-specific early death in elderly LC patients, with all *p* < 0.05 (Table 4).

**Table 3:** The univariable logistic regression analysis of all-cause and cancer-specific early death in elderly LC patients.

Characteristics	All-cause early death			Cancer-specific early death		
	OR	95% CI	*P*	OR	95% CI	*P*
**Sex**						
Male	Ref			Ref		
Female	0.79	0.72–0.86	< 0.001	0.82	0.75–0.9	< 0.001
**Race**						
White	Ref			Ref		
Black	1.2	1.04–1.39	0.014	1.18	1.01–1.37	0.037
Other	1	0.85–1.17	0.99	1	0.85–1.18	0.989
**Marital status**						
Married	Ref			Ref		
Unmarried	1.11	1.02–1.21	0.015	1.1	1.01–1.2	0.026
**Laterality**						
Right	Ref			Ref		
Left	0.95	0.87–1.03	0.244	0.99	0.91–1.08	0.875
Paired site	4.57	0.41–50.44	0.215	5.65	0.51–62.32	0.158
**Primary Site**						
Upper lobe	Ref			Ref		
Middle lobe	0.82	0.67–1.02	0.071	0.86	0.7–1.07	0.19
Lower lobe	0.96	0.88–1.05	0.422	0.95	0.87–1.05	0.318
Overlapping	1.63	1.13–2.34	0.008	1.61	1.11–2.33	0.012
**Grade**						
I	Ref			Ref		
II	1.47	1.22–1.77	< 0.001	1.36	1.12–1.66	0.002
III	2.57	2.15–3.07	< 0.001	2.5	2.07–3.02	< 0.001
IV	3.48	2.75–4.4	< 0.001	3.33	2.61–4.25	< 0.001
**AJCC Stage**						
I	Ref			Ref		
II	1.4	1.12–1.75	0.003	1.65	1.28–2.13	< 0.001
III	2.58	2.26–2.95	< 0.001	3.41	2.92–3.97	< 0.001
IV	6.38	5.64–7.21	< 0.001	8.88	7.7–10.24	< 0.001
**T**						
T1	Ref			Ref		
T2	2.13	1.85–2.45	< 0.001	2.57	2.19–3.01	< 0.001
T3	2.97	2.43–3.62	< 0.001	3.78	3.05–4.69	< 0.001
T4	4.7	4.08–5.41	< 0.001	5.78	4.94–6.77	< 0.001
**N**						
N0	Ref			Ref		
N1	1.54	1.33–1.79	< 0.001	1.57	1.34–1.84	< 0.001
N2	2.35	2.14–2.58	< 0.001	2.59	2.35–2.86	< 0.001
N3	2.37	2.04–2.75	< 0.001	2.75	2.36–3.21	< 0.001
**M**						
M0	Ref			Ref		
M1	3.84	3.51–4.19	< 0.001	4.41	4.02–4.84	< 0.001
**Surgery**						
No	Ref			Ref		
Yes	0.25	0.22–0.28	< 0.001	0.19	0.16–0.22	< 0.001
**Radiation**						
No/unknown	Ref			Ref		
Yes	0.49	0.45–0.54	< 0.001	0.57	0.52–0.62	< 0.001
**Chemotherapy**						
No/unknown	Ref			Ref		
Yes	0.36	0.32–0.4	< 0.001	0.39	0.35–0.44	< 0.001
**Bone metastasis**						
No	Ref			Ref		
Yes	3.02	2.68–3.4	< 0.001	3.34	2.97–3.76	< 0.001
**Brain metastasis**						
No	Ref			Ref		
Yes	3.68	3.16–4.28	< 0.001	4.12	3.54–4.79	< 0.001
**Liver metastasis**						
No	Ref			Ref		
Yes	3.45	2.94–4.05	< 0.001	3.55	3.03–4.16	< 0.001
**Pathological type**						
NSCLC	Ref			Ref		
SCLC	1.39	1.21–1.6	<0.001	1.41	1.22–1.62	<0.001

**Table 4:** The multivariate logistic regression analysis of all-cause and cancer-specific early death in elderly LC patients.

Characteristics	All-cause early death			Cancer-specific early death		
	OR	95% CI	*P*	OR	95% CI	*P*
**Sex**						
Male	Ref			Ref		
Female	0.71	0.65–0.79	< 0.001	0.77	0.69–0.85	< 0.001
**Primary Site**						
Upper lobe	Ref					
Middle lobe	0.78	0.61–0.99	0.045	NA	NA	NA
Lower lobe	0.95	0.86–1.06	0.353	NA	NA	NA
Overlapping	1.36	0.89–2.09	0.155	NA	NA	NA
**Grade**						
I	Ref			Ref		
II	1.27	1.03–1.57	0.026	1.11	0.89–1.39	0.351
III	1.82	1.48–2.23	< 0.001	1.62	1.31–2.01	< 0.001
IV	2.5	1.9–3.3	< 0.001	2.12	1.59–2.83	< 0.001
**AJCC Stage**						
I	Ref			Ref		
II	1.09	0.82–1.45	0.571	1.33	0.97–1.81	0.074
III	1.45	1.18–1.77	< 0.001	1.89	1.53–2.34	< 0.001
IV	2.24	1.83–2.74	< 0.001	3	2.42–3.72	< 0.001
**T**						
T1	Ref			Ref		
T2	1.63	1.39–1.91	< 0.001	1.89	1.59–2.26	< 0.001
T3	2.52	1.98–3.23	< 0.001	2.88	2.23–3.73	< 0.001
T4	2.49	2.09–2.97	< 0.001	2.68	2.21–3.24	< 0.001
**N**						
N0	Ref			Ref		
N1	1.33	1.09–1.63	0.006	1.19	0.97–1.47	0.097
N2	1.51	1.32–1.74	< 0.001	1.43	1.25–1.65	< 0.001
N3	1.38	1.13–1.67	0.001	1.4	1.15–1.7	0.001
**Surgery**						
No	Ref			Ref		
Yes	0.31	0.26–0.36	< 0.001	0.29	0.24–0.34	< 0.001
**Radiation**						
No/unknown	Ref			Ref		
Yes	0.35	0.31–0.39	< 0.001	0.41	0.36–0.46	< 0.001
**Chemotherapy**						
No/unknown	Ref			Ref		
Yes	0.17	0.15–0.2	< 0.001	0.19	0.16–0.21	< 0.001
**Bone metastasis**						
No	Ref			Ref		
Yes	1.78	1.52–2.08	< 0.001	1.81	1.55–2.12	< 0.001
**Brain metastasis**						
No	Ref			Ref		
Yes	3.23	2.66–3.93	< 0.001	3.18	2.62–3.85	< 0.001
**Liver metastasis**						
No	Ref			Ref		
Yes	1.43	1.17–1.73	< 0.001	1.39	1.15–1.68	0.001

### Nomogram construction and validation

**Figure 1:**
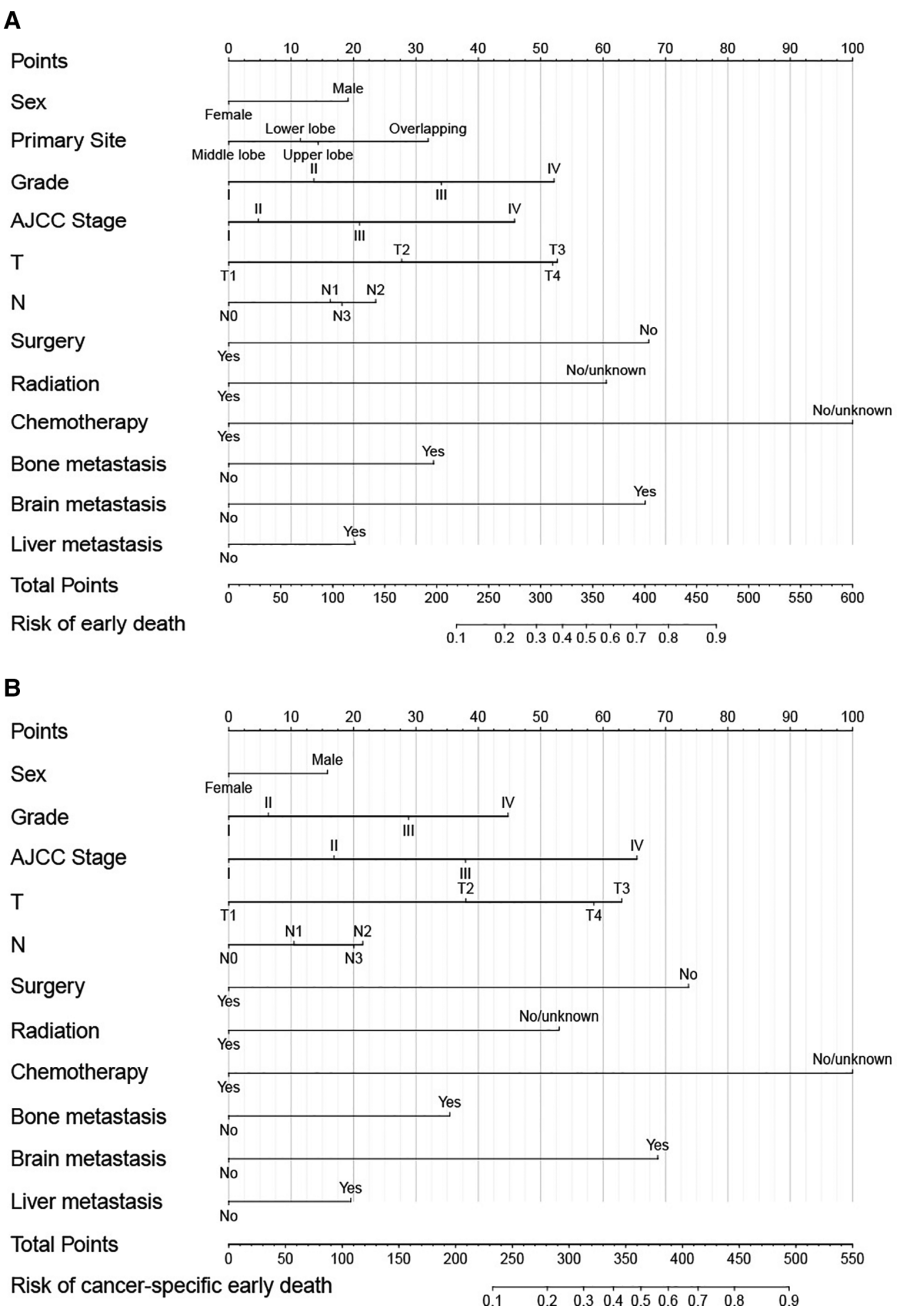
Nomograms for predicting all-cause (**A**) and cancer-specific early death (**B**) in elderly LC patients.

Independent prognostic factors identified by the univariate and multivariate logistic analysis were then included to construct the nomograms. Each variable has the corresponding points, and by adding the score of each variable, the total point and the corresponding risk probability of early death can be obtained. For example, a female patient in the middle lobe with Grade IV, AJCC stage IV, T3, N0, received chemotherapy only, and with bone metastasis only, the risk probability of all-cause early was 35% approximately. As shown in Figure 1A, chemotherapy contributed most to all-cause early death, followed by surgery, brain metastasis, radiation, T, Grade, AJCC Stage, bone metastasis, primary site, N, liver metastasis, and sex. While chemotherapy contributed most to cancer-specific early death, followed by surgery, brain metastasis, AJCC stage, T, radiation, Grade, bone metastasis, N, liver metastasis, and sex.

**Figure 2:**
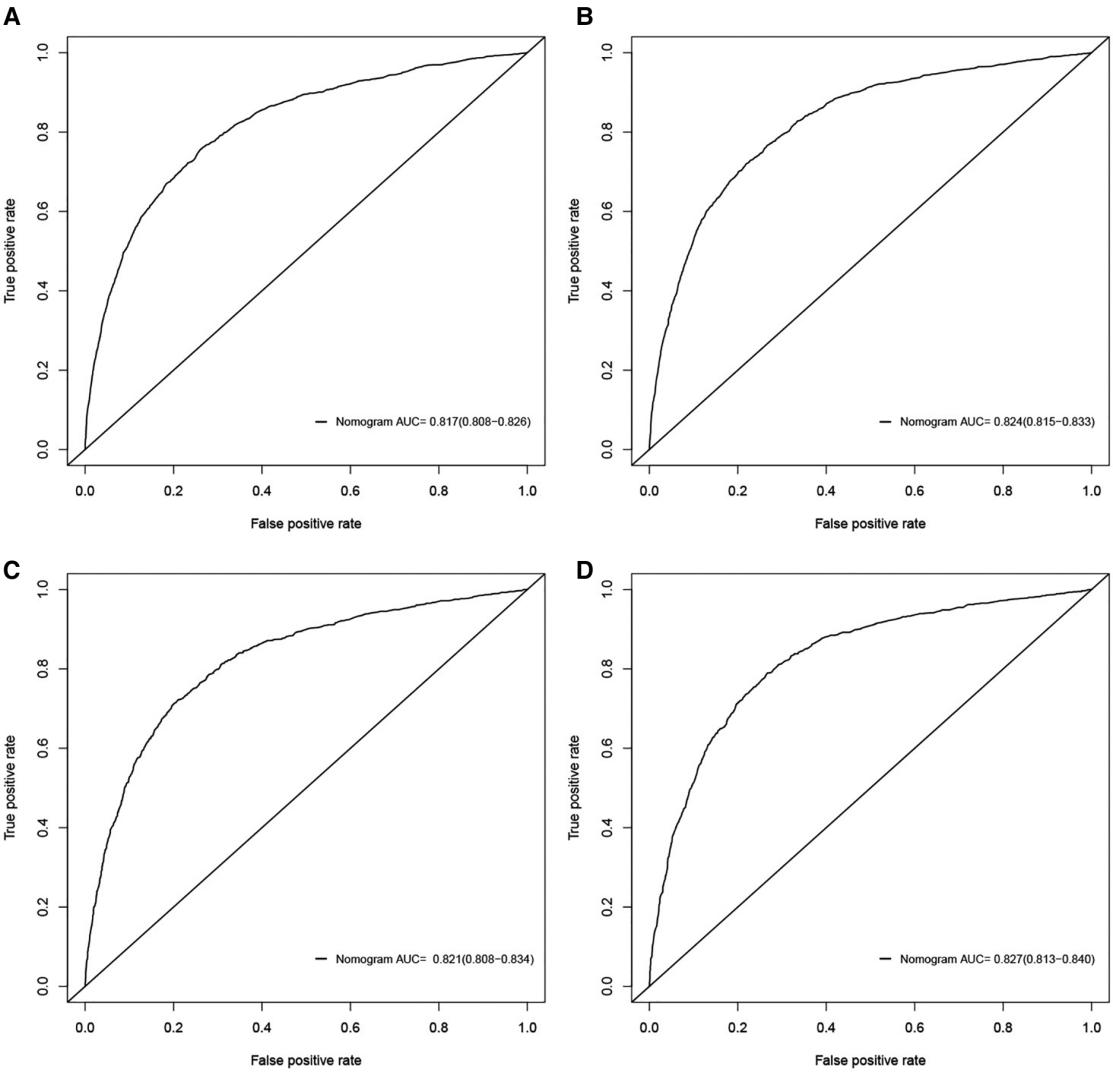
ROC curves for the nomograms in predicting all-cause and cancer-specific early death in the training cohort (**A,B**) and the validation cohort (**C,D**).

**Figure 3:**
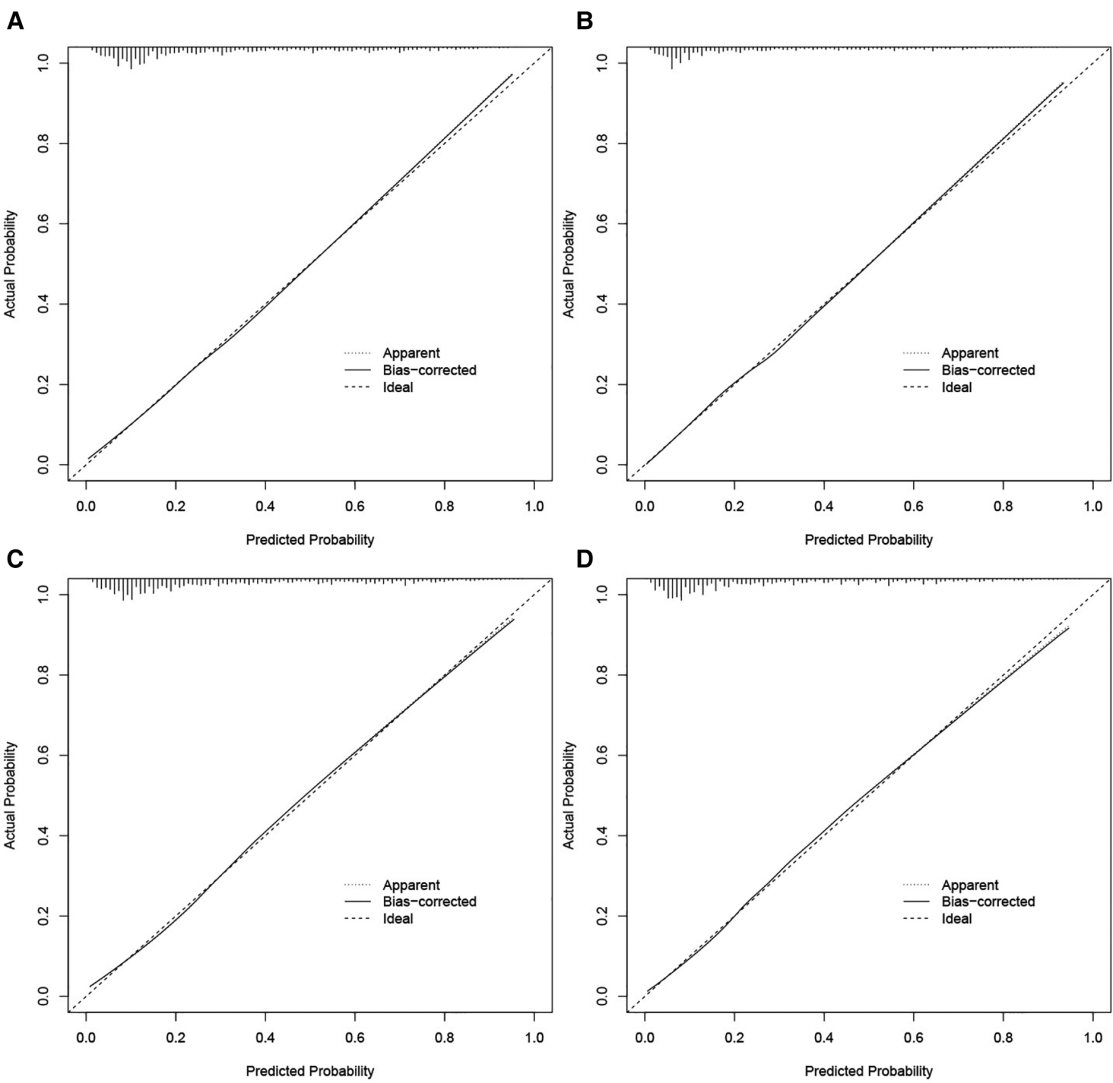
Calibration curves for the nomogram in predicting all-cause and cancer-specific early death in the training cohort (**A,B**) and the validation cohort (**C,D**).

**Figure 4:**
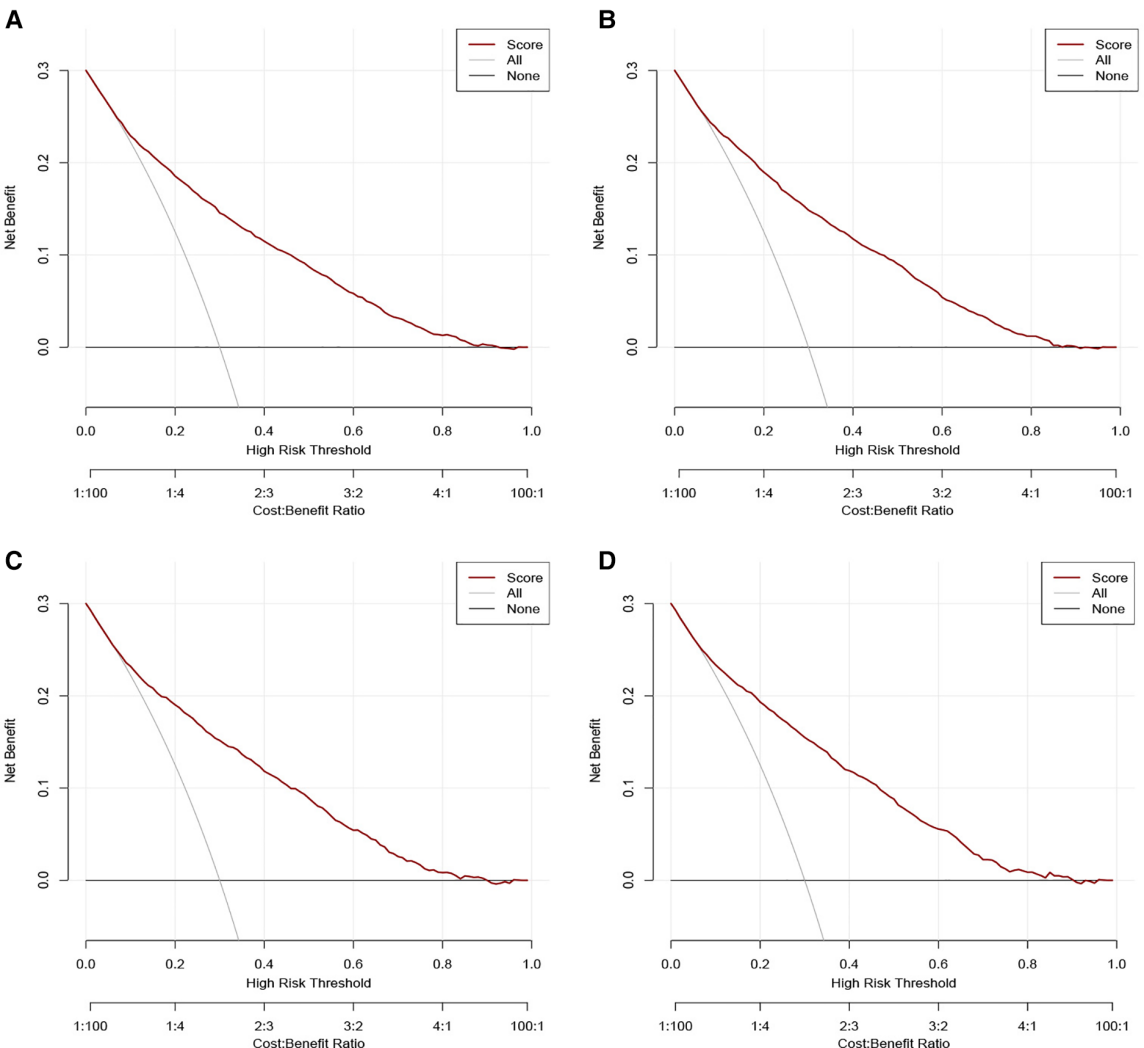
Decision curve analysis (DCA) for the nomograms in predicting all-cause and cancer-specific early death in the training cohort (**A,B**) and the validation cohort (**C,D**).

The validation of nomograms was performed in the training cohort and validation cohort. The results of the ROC analysis indicate that the nomograms exhibited high discriminative ability in predicting all-cause early (AUC in training cohort = 0.817, AUC in validation cohort = 0.821) and cancer-specific early death (AUC in training cohort = 0.824, AUC in validation cohort = 0.827) (Figure 2). The calibration plots of the nomograms were close to the diagonal line revealing that there was a good concordance between the predicted and practical early death probability in the training and validation cohort (Figure 3). Moreover, DCA analysis was utilized to evaluate the prediction performance of nomograms, and the results indicated that the nomograms had good clinical utility in predicting early death probability (Figure 4).

## Discussion

The baseline demographic and clinical characteristics of the patients included in this research were analyzed. Then, risk factors for the early death of elderly LC patients were identified by univariate and multivariate logistic analysis. Besides that, prognostic nomograms for all-cause early death and cancer-specific early death of elderly LC patients were constructed and validated. According to the ROC analysis, calibration plots, and DCA analysis, the nomograms exhibited high discriminative ability and good clinical utility in predicting early death probability.

The evolution of an aging population is a major factor in the increase in LC (10), and until now the prognosis of elderly LC patients remains a challenge worldwide. Elderly patients have increased medical complications, increased risk of toxic side effects and death, reduced tolerance to social stress, emotional, and physical, and reduced physiological reserve (11). Advanced age has been regarded as an independent risk factor for LC (12). Gen Yu et al. found that advanced age is not only significantly associated with poor overall survival but also negatively affected cancer-specific survival (13). Therefore, this research strengthens attention to elderly LC patients and identifies elderly LC patients who are likely to die early.

Consistent with our study, epidemiological studies revealed that male LC patients have a poorer prognosis than female patients (14), and compared with male patients, female LC patients had higher response rates to chemotherapy and longer survival (15). United States research demonstrated that the risk of death in men LC patients was significantly higher than in women LC patients (16). The difference between males and females may owe in smoking because smoking rates are higher in males than females, and susceptibility to tobacco carcinogens varies by gender (17).

Previous studies have investigated a range of models that predict survival in LC patients, but the selected variables were related rare. For example, Yang Zhang et al. included sex, age, smoking history, and other 8 variables to construct web-based nomograms (18). Liang W et al. based on 6 variables to construct a nomogram for predicting survival in LC patients (19). In this research, the logistic analysis demonstrated the contribution of 16 factors, and results revealed that elderly LC patients in higher Grade, AJCC stage, T-stage, N-stage, non-surgery, non-radiation, non-chemotherapy, with bone metastasis, brain metastasis, liver metastasis were related to a high risk of all-cause early death and cancer-specific early death. Moreover, our results showed that chemotherapy was the strongest early-death prognostic factor, indicating that chemotherapy can provide survival benefits and patients should be given it if the patient can tolerate the side effects it.

AJCC stage system which contains T-stage, N-stage, M-stage is a classical model in predicting the prognosis of cancer patients (2). Our research demonstrated that AJCC stage, T-stage and N-stage were independent risk factors for all-cause early death and cancer-specific early death of elderly LC patients. T-stage reflects the tumor size, and tumor size is significantly related to the prognosis in a variety of cancers (20–22). However, whether N-stage is a prognosis factor for LC is still a controversial issue. Studies based on the SEER database revealed that the positive number of lymph nodes was correlated with improved overall survival (23, 24). An Asian cohort study found that the number of lymph nodes was statistically associated with poor prognosis in LC patients (25). Moreover, Gen et al. found that N-stage had no significant effect on the prognosis of LC patients (13). In this research, we found that N-stage was an independent risk factor for the early death of LC patients. However, all of these studies were retrospective analyses. Therefore, more prospective studies and larger cohort studies needed to be conducted to investigate the relationship between N-stage and prognosis in LC. Besides that, the TNM stage does not incorporate other factors such as treatment, histology et al., that may associate with prognosis (26). Therefore, it's of great importance to construct a more comprehensive prognostic model. Nomogram is a precise prognostic model and can incorporate numerous variables, which may help oncologists find high-risk patients and develop better treatment strategies.

From 2010 on, the SEER database begins to contain distant metastasis data, including bone metastasis, brain metastasis, lung metastasis, and liver metastasis. Distant metastases have been found to be the main cause of mortality in LC patients. Bone is one of the most common malignant tumor metastasis sites, and its distinctive microenvironmental status is beneficial to the development and proliferation of tumor cells (27). The previous study found that bone and liver metastases were independent prognostic factors for LC patients (28) and brain metastasis was associated with shorter survival (29), which was consistent with our research. LC patients with brain metastases can lead to neurological dysfunction, seriously endangering the patient's survival (30). Research has found that the overall early mortality of lung cancer was only 27.5% and the mortality of brain metastasis patients up to 44.4%, which indicated the poor prognosis of brain metastasis LC patients.

However, this research has some limitations. First of all, the SEER database does not contain data on molecular pathologic markers. These factors may be effective supplements to the predictive models, and these will be the main part of further studies. Besides that, this is a retrospective study, and the conclusions need to be verified by prospective and larger studies.

## Conclusion

It is of great importance to identify prognostic factors associated with the early death of elderly LC patients and knowledge of predictive nomograms is helpful for clinicians to identify high-risk patients, design individual-based treatments, and develop better treatment strategies.

## Data Availability

Publicly available datasets were analyzed in this study. This data can be found here: http://seer.cancer.gov/.
